# 

**Published:** 1958-01

**Authors:** 


					u- ?
5f
THE
MEDICAL JOURNAL
OF THE
SOUTH-WEST
Incorporating
The Bristol Medico-Chirurgical Journal
January 1958
*-? 73 (i)- No. 267 Price 4/-

				

